# Parental decision making about safer sleep practices: A qualitative study of the perspectives of families with additional health and social care needs

**DOI:** 10.1371/journal.pone.0298383

**Published:** 2024-03-08

**Authors:** Simon Barrett, Jane Barlow, Hannah Cann, Anna Pease, Kate Shiells, Jenny Woodman, Ruth McGovern

**Affiliations:** 1 Newcastle University, Newcastle upon Tyne, Tyne and Wear, United Kingdom; 2 Department of Social Policy and Intervention, University of Oxford, Oxford, United Kingdom; 3 Southampton City Council, Civic Centre, Southampton, Hampshire, United Kingdom; 4 Bristol Population Health Science Institute, University of Bristol, Bristol, United Kingdom; 5 Thomas Coram Research Unit, University College London, London, United Kingdom; Canakkale Onsekiz Mart University School of Medicine, TURKEY

## Abstract

**Introduction:**

Despite a decline in Sudden Unexpected Death in Infancy in the UK since 2004, inequalities have widened with higher rates among families from deprived backgrounds and those known to child protection services. Almost all cases involve parents who had engaged in unsafe sleeping practices despite awareness of safer sleeping advice.

**Objective:**

To understand the perspectives surrounding safer sleep of families supported by statutory child protection agencies, and use behavior change theory to inform how approaches to providing safer sleep advice to these families may be modified.

**Participants and setting:**

We interviewed 14 mothers, 2 fathers and one grandmother, who had recent contact with child protection services in northeast England.

**Methods:**

In-depth, semi-structured interviews, with purposive sampling. The COM-B model (Capability, Opportunity, and Motivation) structured our analysis.

**Results:**

Parents described how anxiety, sleep deprivation, settling infants, illness, and a desire to bond with infants influence their decision making about sleep. Parents valued credible, trusted sources and understanding *how* safer sleep practices protect infants. Responses to questions about ‘out of routine’ situations suggested social pressures surrounding routines and ‘good parenting’ may preclude parents from acknowledging risks and planning for these situations.

**Conclusion:**

Open conversations tailored to the needs of families, focused upon understanding *why* and *when* parent(s) do or do not follow safer sleep guidance seem a promising way of promoting safer sleep practices. Safer sleep discussions with these families are likely to be best delivered as part of wider infant care by professionals who have an established and continuing trusting relationship with parents. While advice and information should be provided by any professional in contact with the family with the necessary expertise, sensitive conversations around sleeping practices, particularly co-sleeping, may be more easily facilitated by professionals where the statutory responsibility for safeguarding is less apparent.

## Introduction

**Fig 1:**
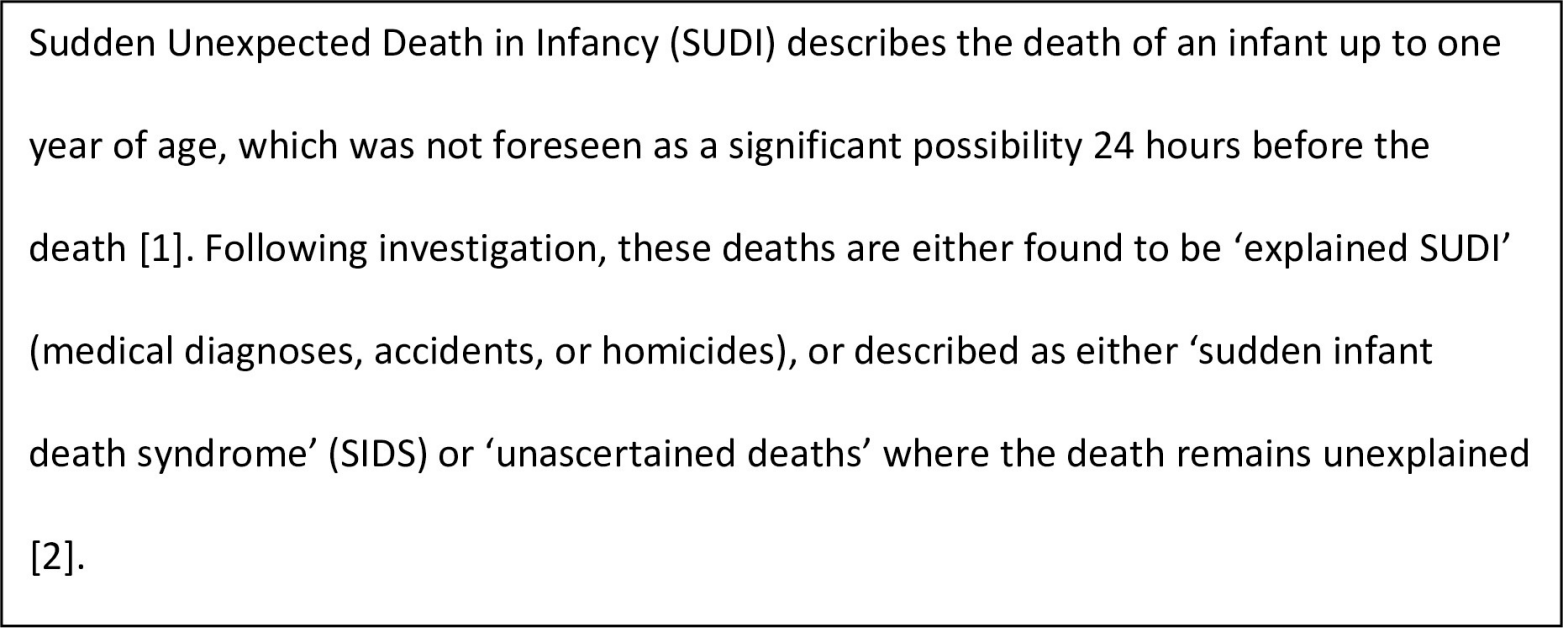
What is SUDI?

Although rates of Sudden Unexpected Death in Infancy (SUDI) (see Fig 1 [1, 2]) have decreased in England and Wales [3], 711 sudden and unexpected deaths of infants occurred between April 2019 and March 2021 [4]. These deaths reveal inequalities in the risk of SUDI, with infants in the most deprived neighborhoods almost three times as likely to die than those in the wealthiest neighborhoods (0.88 v 0.32 per 1,000 live births respectively) [4]. There are several risk factors associated with sudden infant death, including some that are genetic, and others that are environmental and potentially modifiable. These include low birthweight and premature birth; sex of the baby; infant sleeping position; sleep location and area/surface; bed-sharing in the presence of smoking, alcohol, or other drug use by caregivers [5].

Despite an absence of research quantifying the risk of SUDI in families involved with child protection services, evidence indicates an overrepresentation of child protection cases among instances of SUDI. While only around 4% of children in England are referred to children’s social care services [6] where an infant dies suddenly and unexpectedly approximately 30% of these cases will have had contact with child protection services [4]. Additionally, there is an overlap between factors that place children at greater risk of SUDI, such as intimate partner violence and abuse (IPVA), child abuse and neglect, parental mental health issues and substance misuse, and the wider safeguarding concerns that may be present in families in contact with child protection services [7].

The Child Safeguarding Practice Review Panel received 568 serious safeguarding incident notifications for children who had died or suffered serious harm between 2018 and 2019 [7]. Of these, 40 (7%) related to incidents of SUDI, and almost all cases involved parents co-sleeping with their infants in unsafe sleep environments, including those where the parents had consumed alcohol or drugs. In addition, wider safeguarding concerns were frequently present, often involving cumulative neglect, IPVA, and parental mental health concerns [7].

A subsequent review of these cases (The Out of Routine report) concluded that while parents were often aware of, and could cite safer sleeping advice, this advice was not always followed. This was particularly evident in ‘out of routine’ instances—‘unexpected changes in family circumstances immediately before SUDI, in which an infant is placed in an unsafe sleep environment’, and while such ‘out of routine’ situations were present across the full continuum of risk, in families experiencing significant adversity these situations may be even more common [7].

Safer sleep guidance in the United Kingdom consists of advice to place babies on their backs, in a cot in the same room as the parent/caregiver for the first 6 months, with their feet at the end of the cot; keeping the sleeping space clear and level; temperature controlled; never sleeping with baby on a sofa or armchair; and keeping baby smokefree before and after birth. This guidance acknowledges that bed-sharing can happen both intentionally and unintentionally and gives advice on how to do this as safely as possible, and when it should be avoided such as where alcohol, smoking, or drugs are present, or with a baby that was born premature or low birthweight [8].

This safer sleep advice is generally delivered to families by midwives and health visitors (public health nurses who lead the universal Healthy Child Programme for under 5s). Best practice suggests a ‘prevent and protect’ approach [7] where advice is delivered by all agencies who have contact with families, such as Family Nurse Partnerships (intensive support for young first-time mothers [9]), and child protection workers.

The Out of Routine report suggested that interventions that focus ‘solely on giving information are unlikely to produce meaningful change in [a high-risk] population’ and that to ensure future interventions for infants at risk are effective, behavioral change should be used to support the development of safer sleep interventions. Most importantly, families’ experiences, circumstances and perspectives should be considered, and SUDI prevention should be ‘embedded within respectful and authoritative relationship-based safeguarding practice’ [7].

This paper outlines the findings from a qualitative study exploring the perspectives of parents who have been involved with child protection services, to understand the factors that influence their decisions around infant sleep practices, and to inform how safer sleep interventions may be better delivered to this group. We used the COM-B model and Theoretical Domains Framework (TDF) to identify appropriate targets for interventions [10].

### Methods

We undertook in-depth qualitative interviews, with a purposive sampling strategy. We approached agencies and charitable organizations that work with families in northeast England to help us identify families with young children (up to 4 years of age) with recent contact with child protection services. We provided families with an information sheet and explained the research over the telephone to give them opportunities to ask questions before deciding whether to participate.

Our primary contact within each family was the mother, and invitations were extended to include family members who may impact upon sleep decisions and practices. Interviews were offered individually or as a family. Participants were recruited between April and September 2022 and were given a gift voucher as a thank you for their participation.

A semi-structured topic guide was used, with questions based on each of the domains of the TDF (see S1 File). Topics included knowledge, skills, decision making, social influences and environmental contexts. Interviews were carried out by the main author, who has extensive experience of qualitative research and working with vulnerable families. Interviews took place in either the family home or in a residential unit which houses mums and babies in need of shelter, and continued until it was felt that rich data were available for analysis. Interviews were audio recorded and transcribed verbatim.

We used the three components of COM-B model—Capability, Opportunity, and Motivation as the high-level concepts to inform our analysis. The COM-B model identifies that one or more of these components must be modified to bring about behavior change. Capability is defined as the physical and psychological capacity to carry out a behavior. Opportunity refers to the external factors that might influence whether an individual can or cannot carry out the behavior, and motivation is defined as reflective or automatic brain processes that may influence and direct behavior [11]. In this system, capability and opportunity can also influence motivation [11]. For instance, having the knowledge about why a certain behavior is important (capability) or the resources to carry out a certain behavior (opportunity) may mean an individual is more motivated to change their behavior. We built upon this approach using codes derived from the TDF, which builds on the COM-B model and was developed as an integrative framework of behavioral change theories, providing a more granular breakdown of the COM-B components [12].

**Fig 2:**
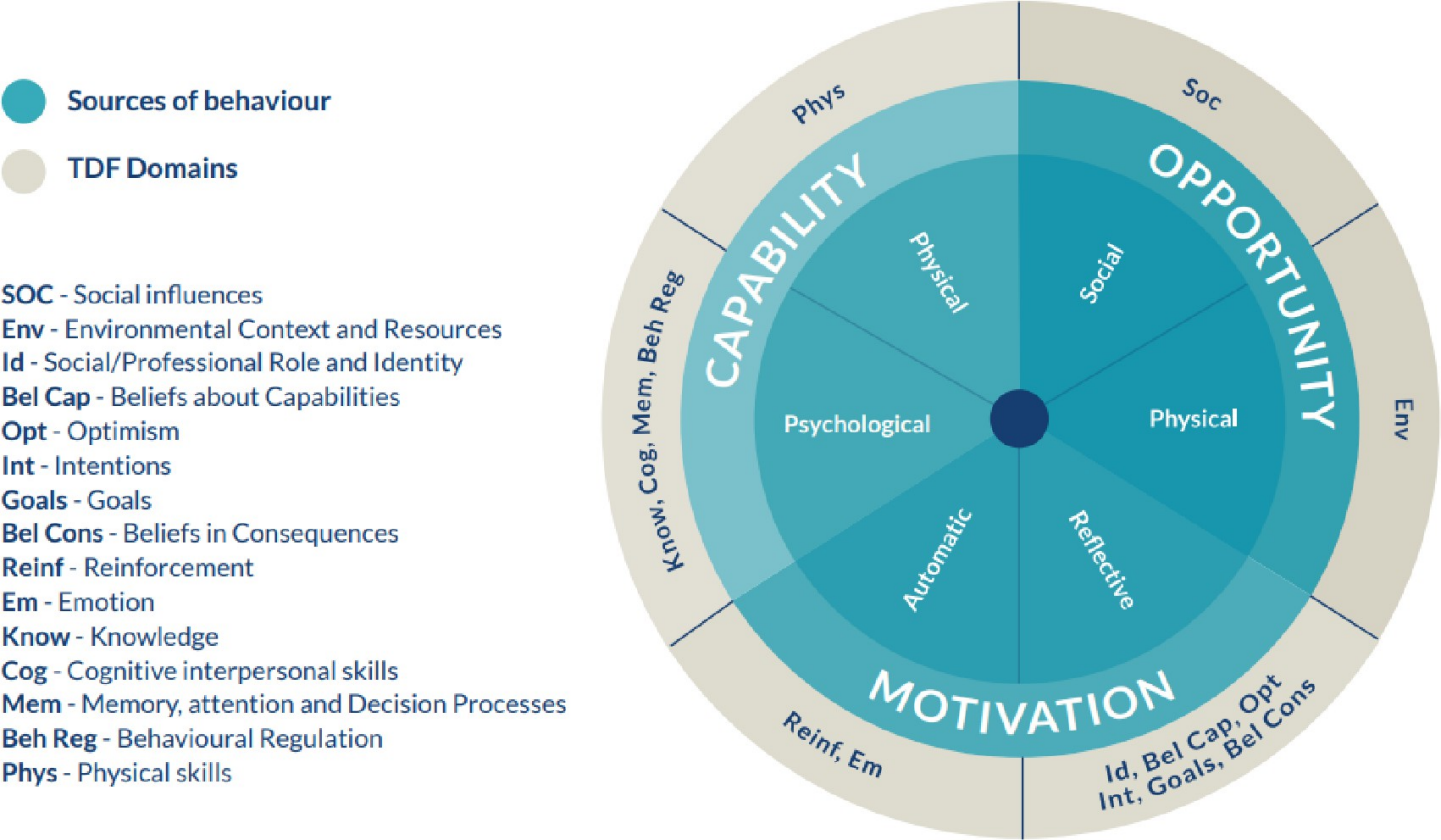
The three components of the COM-B model and the corresponding TDF domains [12].

A social constructionist approach informed our analysis to allow us to uncover the subjective experiences and meanings of our participants [13]. We followed published guidance on using the TDF when analyzing interview transcripts [12], as shown in Fig 2, which also demonstrates how the three elements of the COM-B model align with corresponding TDF domains [12].

The first author coded each transcript according to the domains of the TDF, as well as coding inductively for emerging themes. Transcripts were read by the team, which comprised experienced researchers with expertise in social care research, behavior change, SUDI, and child and maternal health. The coded data were further refined, and emerging central organizing concepts were identified and discussed as a group. An iterative process was adopted whereby early analysis and interpretations of the data informed subsequent interviews and analysis. Authors had access to information that could identify individual participants during or after data collection; all data was kept on secure computer drives and transcripts were pseudonymized. This study was approved by the Faculty of Medical Sciences Research Ethics Committee (2252/16899), part of Newcastle University’s Research Ethics Committee. This committee contains members who are internal to the Faculty. This study was reviewed by members of the committee, who must provide impartial advice and avoid significant conflicts of interests.

## Results

We interviewed 14 mothers aged between 18–37 years from northeast England. Although no inclusion or exclusion criteria regarding ethnicity were set, all participants identified as White British. Six of the mothers described themselves as single, and eight were currently with a partner (in each case the father of the baby). Two fathers and one grandmother took part in interviews and chose to be interviewed alongside the baby’s mother.

For five of the mothers this was their first baby; the remaining mothers had 2–6 children. All families were or had been in contact with statutory child protection services, and had a baby aged between five weeks and 12 months at the time of interview, except for one family whose child who was three years of age. Two babies had been previously removed from the care of their parents by child protection services and recently returned; six of the mothers had older children still in the care of others, and one had recently had her older children returned to her care. None of the infants had a Child Protection plan in place at the time of interview. (Table 1 summarizes mothers’ demographic characteristics).

**Table 1:** Demographic details of interviewed mothers.

Participant	Age of person who gave birth	Baby’s sex	Baby’s birth weight	Baby spent time in Intensive Care?	Number of babies (including this birth)?	Has this child previously been removed from their care?	Older child[ren] removed from their care?	Did person who gave birth smoke tobacco during pregnancy?	Primary caregiver supported by partner?	Partner smokes tobacco?
**1 Emily**	25+	Male	2500g +	No	2–3	Yes	Yes, still in care of others	Yes	No	-
**2 Annie**	21–24	Female	-	No	2–3	No	Yes, still in care of others	-	No	-
**3 Sarah**	Under 21	Male	-	No	1	No	-	No	No	-
**4 Ellie**	Under 21	Female	-	No	1	No	-	-	No	-
**5 Laura **	25+	Male	-	No	2–3	No	Yes, still in care of others	-	No	-
**6 Rebecca **	25+	Female	2500g +	Yes	4+	No	Yes, still in care of others	No	Yes	Yes
**7 Amy**	25+	Male	2500g +	No	2–3	No	No	No	Yes	Yes
**8 Kelly**	Under 21	Male	1750g - 2499g	No	1	No	-	No	Yes	Yes
**9 Danni**	Under 21	Male	2500g +	No	1	No	-	No	Yes	Yes
**10 Jo**	25+	Female	2500g +	No	4+	Yes	Yes, still in care of others	No	No	-
**11 Natalie**	21–24	Female	2500g +	No	2–3	No	Yes, still in care of others	No	Yes	No
**12 Louise**	25+	Female	2500g +	No	2–3	No	Yes, now returned	No	Yes	Yes
**13 Helen**	25+	Female	2500g +	No	1	No	-	Yes	Yes	No
**14 Charlie**	25+	Male	2500g +	Yes	2–3	No	No	Yes	Yes	Yes

The following sections outline the findings of each of the main themes, with pseudonyms used throughout.

### Knowledge

All mothers demonstrated awareness of the main aspects of safety, such as children having their own Moses basket or cot, laying on their backs with their feet at the bottom. For all families, guidance around safer sleep consisted of one or two conversations, sometimes during pregnancy and then in the early days of their child’s arrival. This was often accompanied by a leaflet, signposting to further information online, and invitations to ask questions in the future if they were unsure.

Each of the respondents described the guidance they had received from healthcare practitioners as simple to understand and they especially valued advice when they understood how the practices described were protecting their baby and reducing risks. All respondents cited the risks of suffocation, strangulation or rolling onto their babies while sharing sleeping areas as their main safety concerns.

While some older mothers with multiple children described the guidance they received around safer sleeping as ‘common sense’, ‘nothing I didn’t already know’, and ‘nothing different to with my older two’, there were notable exceptions and gaps in knowledge. Several parents were unaware of some aspects, such as having no bumpers or toys in the sleep space with the baby. One mother felt that she had no knowledge around toys being unsafe during sleep, and struggled to implement this aspect of safer sleep, citing the fact that she had given them to her older children as the reason.

Despite frequent contact with a Family Nurse, Ellie felt that she had not been advised on how to put her baby safely to sleep in a cot.

*I never got any advice on what you should do with the babies in a cot*. *I still haven’t got a clue now*. (Ellie, 19, 1 child).

Although some mothers felt that because they had older children there was an assumption from practitioners that they did not need any guidance, Louise described how both her child protection worker and her health visitor had told her things she did not know or had forgotten since she had children over a decade ago. However, when she experienced anxiety around safer sleep, she chose to find solutions by searching online, rather than discussing this with practitioners.

*You know when they’re first born*… *I used to keep checking on her all the time in case the blanket went over her face or anything like that*. *So*, *[I went online and] when I found the baby sleeping bag it made me feel so much better*. (Louise, 36, 3 children).

Other younger mothers also talked about their use of the internet and social media as a primary source of information around safer sleeping.

*I Google it*, *then I’ll read every single one*, *think which one is the best and I’ll just click on that one*. (Annie, 24, 2 children).

Conversely, where a healthcare professional had pointed to a credible source, it did not always seem to be useful or relevant.

*When I was pregnant my midwife said if there is anything you want to know about safe sleep and stuff go on the Lullaby Trust website* [8]*…I think possibly I might have had a quick glance*, *but to be honest it was all the basics what I already knew*, *so I had a glance when I was pregnant*, *but I have never been on since*. (Amy, 34, 3 children).

### Capability and routine

Developing a daily routine around sleep was seen by mothers as important for their babies, and often this routine was the primary focus for parents. For many, this was particularly challenging due to disruptions in their lives associated with their mental health, or experiences of domestic abuse for example.

The ability to have children in a good routine was seen as evidence of good parenting, and something they felt demonstrated their capabilities as a parent, even in challenging circumstances such as addiction. Although safer sleep practices were seen as important, they were frequently discussed in terms of how they were incorporated into daily sleep routines.

*When they’re first born*, *in the Moses basket*, *you tend to not put a blanket on them*. *I always use the grow bags*. *And having no toys in with them… he’s been in his [own] room since he was six months old*. *I’ve done that with all three of mine*, *they’re all into their own rooms by six months*. *Even though I was addicted to drugs*, *their routines were always the same*. *I’ve always been big on they need routine*, *from being a baby*. (Emily, 25+, 3 children).

Emily described how although she was addicted to drugs at the time the removal of her two older children by child protection services, she felt this decision was not connected to her drug use or their lack of routine, something that she was proud that was ‘read out in court’ proceedings.

However, for some mothers, getting their baby to sleep each evening remained challenging, whether due to the needs of their child, or the family’s living arrangements. Laura described how the routine she had in place from an early age was disrupted when she and her baby had to leave the family home and stay in a mother and baby unit due to IPVA.

*Before I moved [to mother and baby unit]*, *[son*, *aged 1] was in a routine from 11 weeks old… I never felt better in my entire life*. *I was so proud of myself*, *telling everyone*, *"I’ve got my baby in this routine*,*" literally bath*, *bed and he slept all night until 7*:*00 in the morning*. *I felt great … Then I moved there*, *he was like 8 months*, *it just all fell to pieces*. *Now I’m struggling again … [I’m] just in the habit now—which is absolutely awful—of just leaving the telly [TV] on for him*, *and I’ll put my headphones in—it was driving me insane because I could hear him cry…I need to get out of that habit because I know it’s not healthy*. (Laura, 25+, 3 children).

The disruption not only affected Laura’s interaction with her baby, but meant that as well as their sleep routines being disturbed, safer sleep practices were compromised. Describing an overwhelming sense of depression, as well as cramped sleeping spaces and a noisy environment within the unit, she no longer felt able to adopt the healthy routines she previously had.

For other parents, a routine was not possible because they felt they lacked the ability to settle their baby in their own sleeping space. This approach often led to potentially unsafe practices.

*[Baby] won’t sleep any other way*, *she’s either on my chest or across me*, *and I’m watching telly*. *And then I have to move very carefully*. *I usually just sit there like a zombie [while baby sleeps on her chest]*. (Rebecca, 25+, 6 children). *Even though she’s been told that when [baby] sleeps*, *you sleep*, *kind of thing*, *she won’t*. [opting instead to attempt to sit awake all night with the baby on her chest]. (John, 43, 2 children).

Others also struggled to settle their babies or establish routines due to the needs of their child (such as teething or illness) and described modifying their approach such as being awake through the night to comfort their sick baby or sleeping on the floor beside the cot to be closer to them.

Many mothers reported feeling confident enough to share both their knowledge and their expectations about routine when the baby was in the care of others, such as grandparents. However, for all families such circumstances were infrequent due to the Covid-19 pandemic and the associated restrictions on travel and social gatherings in place around the time their babies were born.

### Opportunity and resources

While all families were from deprived socio-economic backgrounds, this was rarely identified as a factor affecting their ability to adhere to safer sleeping advice. All but two families had secured the equipment for sleep that they felt they needed before their baby’s arrival. These were purchased new, or from local second-hand sources or received from family and friends. One family, who were homeless when the mother was 22 weeks pregnant, had been helped by a child protection worker to find a property, and another had sourced baby equipment, including a cot, from a local charitable organization recommended by their health visitor. All families were supported by child protection services or third sector groups, and in some cases, several mothers were helped to find suitable accommodation when fleeing violent relationships; however, they did not appear to rely upon child protection services or third sector groups for the provision of baby equipment. In cases where mothers were provided with accommodation in refuges or mother and baby units, which included cots and beds, they did not rely upon help from these organizations to source equipment when returning to their own homes.

### Motivation–bonding and anxiety as motivation to co-sleep

Four mothers described taking the decision to share a sleep surface with their babies, in each case motivated by fear and anxiety. Ellie described how awareness of cot death and potential harm to her baby left her frightened and unable to cope, a situation which then led to her co-sleeping.

*I ended up having to stay with my mum because I was panicking*, *obviously*, *cot death—people got it drilled into my head*, *“You can’t do this*, *you can’t do that” so it was scaring me*. *I couldn’t cope being a new mother*. *It was hard*. *I felt like I had no one to help me*. *The resentment towards my daughter was just getting worse*. *So that’s when I just decided what happens if I share a bed with her*? *So*, *I just tested it out*. *I got a lot more sleep and she got a lot more sleep and we just felt better the next day*. *It got to the point where she had to actually sleep in my bed so I can feel her breathe*. *I felt better*. *I felt more safe and less anxious because she was there right next to me*. (Ellie, 19, 1 child).

For one mother with a history of substance use, her decision to co-sleep was motivated by anxiety, exacerbated by complications her son faced at birth. This decision was also contrary to her initial intentions.

*[He] was born not breathing properly…And he was addicted to Tramadol*… *showing massive withdrawal*. *He was shaking*, *wasn’t breathing*, *he was really upset*. *I was one of those people who was “He will not be in my bed”*, *I was very adamant of that*. *I thought I knew what I wanted to do*, *but I think as my anxiety grew*, *it seemed to get way worse… I brought him in [to my bed]*… *I just done it; I didn’t give anyone else an option*. *At that point I was extremely anxious about being away from him*. *I think it was first for a nap… and it just progressed*. (Charlie, 36, 3 children).

Another took the decision co-sleep prompted by a reflux episode at aged four months where baby had begun to choke. This decision was motivated by anxiety, exhaustion, and the need to have her daughter close, and despite problems with alcohol use, she felt the risks associated with co-sleeping were acceptable.

*Alcoholic behavior… I think you persuade yourself that your drinking is not that bad*. *“Well*, *it’s only a little bit of wine*.*” Or…the thought wouldn’t even cross my head*. *I would have just been “It’s a risk I’m willing to take*.*”* (Helen, 31, 1 child).

Each of the mothers who shared a sleeping space with their children also attempted to mitigate the known risks with modifications to their shared sleeping area. These included using cushions on the bed and floor, and changes to sleeping positions, and were often accompanied by accounts of being light sleepers and being more responsive to their baby while co-sleeping. These decisions were described as instinctive and were not discussed with practitioners. While motivated by the desire to increase safety, in some circumstances these modifications may have in fact made conditions more unsafe.

*[W]hen she was a teeny baby*, *she would sleep on a pillow*, *which when you put her on it*, *she sinks into it*. *So*, *it cradles her*, *so she can’t move anywhere*. *And then I would just cuddle into the pillow*, *and into her*. (Ellie, 19, 1 child).

Mothers also described how sharing a sleeping space with their child had good outcomes for their family, which reinforced their motivation to continue with this practice.

*She did seem to get better sleep*. *She settled a lot better*. *I think just knowing that she was next to us*, *settled her down*… *I got better sleep too… She’s from then been a really good sleeper*. *It was so lovely falling asleep with her there and waking up with her there*…*to have that time*, *especially when I got sober*, *to have that time of before I go to sleep*, *and then waking up in the morning and seeing her there*, *it’s precious*. *That time is really important to us*. *It’s one of my favorite points of the day*…*it’s added to the relationship that we have together as well*. (Helen, 31, 1 child).

Similarly, Ellie who had initially found bonding with her daughter difficult due resentment and the trauma she had experienced in a violent relationship with the father, described an improved relationship with her baby because of co-sleeping.

*[Sharing a bed] brought me closer to her*. *I know a lot of people say*, *“Oh*, *the love that you have for your baby is like no other*,*” where I despised her… I still loved her in my heart*, *but in my head*, *I didn’t*, *because of everything that’s happened*. *So*, *sharing the bed with her*, *made me love her in my head as well as in my heart*. *And now we’re just inseparable*. (Ellie, 19, 1 child).

Charlie described how sharing a bed with her son brought benefits to them both, and although doing so created some anxiety due to the risks involved, overall, this anxiety was easier to manage when she was co-sleeping.

*Yes*, *it is definitely a me thing*. *[Son*, *5 months] loves being beside us and he sleeps longer when he’s beside me—he definitely gets a good sleep when he’s in his cot as well*… *but the anxiety seems worse about him being away from me than being with me*. (Charlie, 36, 3 children).

Although none of these mothers chose to disclose co-sleeping practices to professionals or even their friends, they often found validation when reading on the internet that others were co-sleeping.

### Relationships with professionals

Those that described sharing a sleeping space with their children also reflected upon their decisions to withhold this information from practitioners who worked with the family. For one this was associated with her alcohol use and being ‘sensitive to criticism’ (Helen), and for another it was a similar ‘fear of judgement’ and the fact that nobody else ‘needed to know’ (Charlie). Along with others, Charlie felt that because she had older children there was an assumption from practitioners that she did not need any guidance. This was compounded by a tendency to *‘not to ask [for advice]*, *because a lot of social workers will look at you as if you don’t know how to parent your child’*. (Charlie, 36, 3 children).

Despite the intense support provided by her Family Nurse, Ellie also chose not to discuss her decision regarding sleeping arrangements, but rather let her Family Nurse assume that ‘safer’, seemingly more acceptable practices were in place.

*We got onto the conversation*, *but I didn’t tell her I was sharing a bed with her because she’d be*, *like*, *“No*, *she needs to be in her own bed*. *These are the risks*, *don’t do it*.*”*. *Luckily*, *I still had a Moses basket next to my bed and it had all her old blankets and then it sort of looks like she’s sleeping in that*, *but actually*, *she wasn’t*. *Whatever she was saying I was happy with it because she didn’t question where she’s sleeping*. *She just*, *kind of*, *automatically thought she was in a Moses basket*. (Ellie, 19, 1 child).

Conversely, some families–none of whom described any instances of co-sleeping–appeared to have open conversations with the practitioners, in particular health visitors, who were supporting them. This resulted in confidence to ask questions, as well as what appeared to be a desire to ‘comply’ with the advice they were receiving.

*She [midwife] wasn’t happy*, *so we had to go get [the mattress in the Moses basket changed] straight away*. *She was really unhappy with it*, *and then came back the next day it was changed*. *She was really surprised*. (Sean, aged 19, 2 children).

For some, this guidance was perceived as instruction, and families valued the practical guidance and support provided by practitioners. When delivered in this way, families reported an increase in both their capability and motivation to implement safer sleep practices.

*She [child protection worker] used to come around once a week and show us stuff*. *I never got told anything like that when I had my other children*… *everything was all new to me… we did everything that we got told to do and social services couldn’t be happier*. (Rebecca, 25+, 6 children).

Often families saw the health visitor as a trusted person, and they were frequently the go-to person for specific questions on sleep or to check something is safe: *‘mostly the health visitor or me mam’* (Amy, 34, 3 children), and in some cases of instead of their own family.

*My mum does try to give me advice*, *but I always double-check with my health visitor as well*, *just because my mum is very old-fashioned in the way that she raised me*. (Danni, 19, 1 child).

However, others suggested that the advice health visitors gave was simply a brief conversation, giving out leaflets, or *‘more of a passing comment’* (Charlie, 36, 3 children).

Table 2 provides a summary of key findings mapped onto the COM-B model and the corresponding TDF domains.

**Table 2:** Summary of key findings mapped onto COM-B and TDF domains.

COM-B Behavioral component	TDF domain	Key findings	
Capability Psychological	Skills	Several parents described being unable to settle their baby and would choose to co-sleep or try to stay awake all night	
	Knowledge	The majority described sleep practices consistent with guidance and being able to ask questions of practitioners; some reported that safer sleep advice was not followed, and was not discussed with practitioners; Some parents with older children felt that advice generally did not go into detail about updated guidance	
	Memory, attention & decision processes	Understanding risks and why practices protected infants was seen as an important part of safer sleep messaging	
Opportunity Social and environmental	Social influences & environmental context & resources	Some parents report informing their own parents about updated guidance. The majority were able to source sleep equipment	
Motivation Reflective	Beliefs about capabilities	The majority felt they had autonomy; however, several parents described how their decisions were influenced by social workers, and that they were keen to demonstrate this ‘compliance’ with safer sleep guidance	
	Optimism	Mothers who reported co-sleeping relied on practical measures such as pillows to prevent them from rolling onto their babies, or babies from rolling out of bed, and adjusting their own sleeping position or style to make it ‘safer’ for their baby	
	Beliefs about consequences	Many parents described using a cot and never co-sleeping due to fear of harming their baby, and that getting the baby to sleep in a cot was an important aspect of establishing good habits and routines; Several parents reported propping up mattress to elevate the baby’s head when they were ill	
		Several parents described how they felt more able to monitor their baby while co-sleeping; those mothers who did co-sleep described themselves as light sleepers or hyper aware of their babies when sharing a sleeping space with them	
	Goals	Decisions to co-sleep were framed around being able to take better care of their baby; none of the mothers were breastfeeding	
Motivation Automatic	Emotion	Those parents who co-slept reported that doing so gave them a closer bond with their baby, reduced their anxiety, or provided them with better sleep	

## Discussion

Our findings suggest that capability and motivation are two key factors of the COM-B framework that need further attention in terms of supporting safer sleep practices within this population of parents.

### Capability

In terms of capability, although knowledge and routine were two key elements of parents’ ability to keep their infant’s safe, some participants described routes to knowledge that were not through the professionals with whom they had contact. The findings of this study suggest that for this group of families, despite frequent contact with services who deliver safer sleep messages (e.g., Family Nurse who should see a family up to 64 times before a child is two) and being given information from a variety of sources, there were gaps in their knowledge.

In addition, safer sleep routines were harder to implement for parents who had experienced substance use or violence and who were experiencing high levels of anxiety. A lack of knowledge, skills or confidence, for example in settling infants, may have also contributed to unrealistic and unsustainable goals, such as attempting to stay awake all night. Our findings suggest that parents’ experiences and instinct may therefore be more influential on parental behavior than the guidance they receive.

As with previous studies, for some families we interviewed, education and guidance on safer sleep influenced how and where infants slept [14–16]; however, this approach to giving information can also be ineffective for some, especially when information is delivered in a condescending, didactic style, without opportunities to ask questions [17]. The findings of this study are also consistent with previous research which indicates that where professionals do not take the time to explain safer sleep recommendations, for example where it is not the parent’s first child, parents may be left unsure about the most up-to-date recommendations [17]. Similarly, in line with existing research, some parents in this study were considerably less likely to follow recommendations if they did not understand the protective mechanisms, and when they lacked skills or confidence to act on the information that they received [14, 17, 18].

### Motivation

In terms of motivation, the sleep practices of parents we interviewed were often driven by a range of goals in relation to themselves or their infant including their own and/or the child’s need for sleep; to address emotional needs such as bonding with their baby; the need for emotional and physical closeness; or to reduce their own anxiety. These motivations were described as instinctive, and their attempts to mitigate the known risks sometimes involved the use of techniques that may have been less safe. The benefits that families perceived from co-sleeping reinforced their motivation to continue doing so.

While many mothers were aware of safer sleep guidance, the application of this in real life situations was not always possible for them or did not correspond with their own motivations. Providing information about SUDI has been demonstrated to make some parents feel more anxious, without necessarily leading to any positive changes in their behavior [20], and for some families in this study, increased anxiety appeared to be a motivator to co-sleep and to adopt practices that may have increased risk. As such, the provision of information without additional support may have adverse, unintended consequences for some families.

Taken together, these findings provide insight into why providing information alone is insufficient to affect positive changes in behavior. Information may therefore need to be provided as part of a motivational and coaching approach, which supports families by exploring their capabilities and motivations (for example the desire to bond with their baby) and how these are enacted in day-to-day life. Such an approach should also explore alternative methods by which capabilities may be enhanced and motivations may be attained.

### Implications for practice

Existing research indicates that parents are generally more likely to follow advice from family, peers or the internet than professionals [16, 19–22], and that risks may increase, and safer sleep may become less of a priority when children stay away from home [15, 17] or when following family ‘traditions’ with regard to infant sleep [19]. However, our findings suggest that some families were able to resist these trends. Not only were some parents able to implement safe sleep practices despite contrary advice from family members, but the guidance from professionals was important to them, and was frequently passed on to other caregivers. This would suggest that within this group, trust and positive relationships with professionals is a key factor in implementing safer sleep practices.

In contrast, the relationship that other families had with the professionals delivering safer sleep guidance was important in terms of their unwillingness to discuss some of the sleep practices they adopted. For example, where families decided to co-sleep, they were aware of the potential risks of doing so, and their decision to co-sleep was positively reinforced by perceived improvements in bonding, reduced feelings of anxiety, or better sleep for themselves and their baby, but in all cases this decision was withheld from their family support workers. None of the families we interviewed described discussing co-sleeping as an option with any professionals with whom they had had contact, and these may therefore represent missed opportunities to discuss safer ways of co-sleeping for this group of families. These findings highlight the importance of families being able to sufficiently trust at least one practitioner, to enable them to have an open and honest discussion about their infant sleep practices. It may also suggest that professionals may need to ask about co-sleeping, where they are able to provide evidence-based guidance and practical support to reduce potential risks to the infant. Given that suffocation is a major concern for parents, professionals may find it useful to incorporate advice about reducing the risks of suffocation into conversations about safer sleep, while being mindful of misconceptions surrounding the causes of SUDI.

Our findings indicate that efforts to reduce SUDI among families with a child protection worker should go beyond safer sleep ‘messaging’ and simple information giving. For example, using open and meaningful conversations between parents and practitioners about infant sleep, that are embedded within wider discussions about infant and maternal care such as settling and expectations around carers being able to stay up all night comforting infant. Techniques such as motivational interviewing may be a useful way of having such conversations with families engaged with welfare services [23, 24]. Motivational interviewing entails a “collaborative conversation style for strengthening a person’s own motivation and commitment for change” [25] (p12) and has promising evidence of effectiveness within this population when used alongside other programs [24, 26]. Motivational interviewing may also be effective in improving engagement with services, as well as outcomes among parents who use substances, who have depressive symptoms, and who have a history of child maltreatment [27, 28].

These conversations may, however, be difficult because of fear that families often feel in relation to child protection services, and difficulties with trust due to adverse early life experiences [29]. Our findings indicate that for this group of families, health visitors were often the most trusted practitioners. This suggests that such while safer sleep advice and information should be provided by any professional in contact with the family with the necessary expertise [30], sensitive conversations around sleeping practices, particularly co-sleeping, may be better facilitated by professionals who are not also responsible for child protection matters within the family. Programs of intensive home-based support, embedded within universal health systems but delivered by non-statutory services, may provide suitable opportunities to facilitate open, motivational conversations and provide continuity of care. The Maternal and Early Childhood Sustained Home visiting program, for example, provides additional support to families by healthcare professionals, and has been demonstrated to have positive outcomes in terms of knowledge of SUDI risks [31], as well as parental responsivity and self-efficacy [32].

Open conversations around risk may also be difficult where professionals operate in risk-averse systems, which prevent them from being able to manage rather than avoid risk. There may be opportunities within system infrastructure to consider and modify organizational culture for working with ‘at risk’ families. For example, in England, Integrated Care Boards may consider approaches which provide blueprints for service/procedureswhen working with families considered vulnerable or with a social worker. Further, the establishment of Multi-Agency Child Protection Units in England—integrated and co-located multi-agency, multi-professional teams staffed by experienced child protection practitioners–in every local authority area, as recommended by The Child Safeguarding Practice Review Panel [33], may also help to facilitate the necessary changes in organizational culture to support practitioners to manage rather than avoid risk. These units have the potential to lead on organizational culture change and skill development within child protection services and ‘provide workers with the supervision and support they require’ in order to assess and manage risks to children more effectively [33] (p100). The use of motivational interviewing may also be a helpful strategy for helping to change organizational culture in child protection settings [34].

The findings of this study also suggest that the use of the term ‘out of routine’ may not be helpful to practitioners in terms of characterizing unusual situations where the infant may be at increased risk. Many parents regarded the term as being critical of them as a parent, and thereby this may not be useful to facilitate open conversations about situations that are atypical. Acknowledgment that such disruptions and ‘busy nights’ are normal, for example, may be more effective.

## Strengths and limitations

This paper uses empirical data to understand the perspectives of an under-researched population, and the factors which influence their decision making in terms of infant sleep practices. The use of the COM-B model and associated TDF domains are a strength which provide behavioral insights to this complex problem and may improve the prospects of interventions being successful [35].

When discussing sensitive issues, especially among families who have experienced intense scrutiny and surveillance of their parenting skills, social desirability to demonstrate compliance with guidance and being a good parent may have resulted in families feeling unable to disclose any potentially risky practices they adopted or were contrary to guidance they received.

Although the families we interviewed were often experiencing problems such as substance use, poor mental health, and IPVA which may be similar to families where elevated risk has previously been identified, we were not able to ascertain whether the infants in families we interviewed were at greater risk of SUDI. The parents we interviewed were supported by third sector organizations and/or child protection services at the time of interview, meaning that they were potentially not at the point of highest risk. Our sample, although diverse in age and parity, were heterogeneous in terms of ethnicity and were recruited from one region in England, which will impact the generalizability of these findings. For example, our findings do not represent any non-White ethnic groups where cultural practices around co-sleeping may be relevant.

## Conclusion

The findings of this study suggest that capability and motivation are key areas that drive behavior in terms of infant sleep practices, and that these domains may provide significant opportunities to influence behavior. Open conversations tailored to the needs of families and focused upon understanding *why* and *when* parent(s) do or do not follow safer sleep guidance appear to be a promising way of promoting safer sleep practices. Safer sleep discussions with these families are likely to be best delivered as part of wider infant care by professionals who have established an ongoing trusting relationship with the parents, and possibly where statutory responsibility for safeguarding is less apparent. The use of motivational, conversation-based approaches may enable practitioners to have a greater influence on behaviors, particularly where they are able to acknowledge common situations in which risks may be increased, and support families with planning for safety during those times. This approach is likely to require additional training and support for professionals and a change of organizational culture to allow them to manage and hold risk more confidently.

## Supporting information

S1 File Interview topic guide. (DOCX)
