# Supplementary material for: Parental decision making about safer sleep practices: A qualitative study of the perspectives of families with additional health and social care needs
Source: PLoS One. 2024 Mar 8;19(3):e0298383. doi: 10.1371/journal.pone.0298383 (PMC10923434; doi:10.1371/journal.pone.0298383)
Supplement: S1 File — (DOCX) [file pone.0298383.s001.docx]

Supplementary Materials – Interview topic guide

1. Background - family life/situation? ages, no. of children, partner, extended family, others sharing house, support in place etc. **social role** –influence they have upon sleep?
2. **Knowledge** - what you know about safer sleep guidance, experiences of advice?
3. knowledge of risk factors; sources of information
4. Were they seeking advice, or given without asking?
5. Did this change if they had previous children?
6. **Beliefs about consequences** – what would happen if you didn’t follow guidance?
7. **Optimism, reinforcement** – what are the (dis)advantages of following advice?
8. **Intentions/Goals** – what are your motivations to follow/not guidance? Do you set goals to follow all/some, on most days/until a certain age etc.
9. **Skills** – what practices do you employ for managing sleep?
   1. how they acquired practices– previous experience, social media, trials
10. **Beliefs about capabilities** –confident about [consistently] managing sleep?
11. ability to introduce sleeping routines/respond to babies needs when crying/manage competing responsibilities e.g. other children, partner, work.
12. Self-efficacy – confidence to implement safer measures
13. **Behavioral regulation** - confident to maintain practices out of routine?
14. What happens in out of routine situations such as visiting others/parties
15. **Decision process -** What informs your decisions relating to safer sleep practice?
16. parent’s needs, baby’s/other family member’s needs
17. **Environmental context and resources –** are there things about where you are living or about your situation that impact on safer sleeping?
18. Presence of risk factors – overcrowding, smoking, substance use
19. Other issues like lack of resources, baby equipment/furniture/bedding etc.
20. **Social influences** – are there other people who impact upon the sleeping practices?  Friends’ or parents’ influence, other children; IPVA, substance use?
21. **Emotion** – how do you feel when you are not able to follow the guidance?
22. feelings of remorse or guilt etc.
23. Does this impact on future decisions?
24. Does this make asking for support or discussing with practitioner difficult/fear of consequences etc.?
25. How do you feel safer sleep guidance could be delivered better?
26. what would have helped to follow the advice? e.g., tips to help babies settle, would you have looked at those/tried any of them out?
27. Terminology, medium, person delivering message, language?
28. Resources needed, and ideas about how to improve information, skills, and change behaviors?
